# Mortality from Cancer in England, as Regulated by Sex

**Published:** 1847-02

**Authors:** 


*Mortality from Cancer in England, as regulated by Sex.*

| Year of Report. | Total fatal Cases of Cancer. | In the Female Sex. | In the Male Sex. |
|---|---|---|---|
| 1838 | 2304 | 1717 | 587 |
| 1839 | 2549 | 1924 | 625 |
| 1840 | 2238 | 1656 | 582 |
| 1841 | 2215 | 1692 | 523 |
| 1842 | 2356 | 1757 | 599 |
| Total, | 11662 | 8746 | 2916 |

*Ibid.*

